# Cows with diverging haplotypes show differences in differential milk cell count, milk parameters and vaginal temperature after *S. aureus* challenge but not after *E. coli* challenge

**DOI:** 10.1186/s12917-024-03996-y

**Published:** 2024-05-15

**Authors:** Katharina Müller-Langhans, Lisa Oberberger, Yury Zablotski, Susanne Engelmann, Martina Hoedemaker, Christa Kühn, Hans-Joachim Schuberth, Holm Zerbe, Wolfram Petzl, Marie Margarete Meyerholz-Wohllebe

**Affiliations:** 1 Clinic for Ruminants With Ambulatory Clinic and Herd Health Services, Center for Clinical Veterinary Medicine, Ludwig-Maximilians-University Munich, Sonnenstrasse 16, Oberschleissheim, 85764 Germany; 2 Institute for Infectious Diseases and Zoonoses, Ludwig-Maximilians-University Munich, Sonnenstrasse 24, Oberschleissheim, 85764 Germany; 3 Technical University Braunschweig, Institute for Microbiology, Inhoffenstrasse 7, Brunswick, 38124 Germany; 4 Helmholtz Center for Infection Research, Microbial Proteomics, Inhoffenstrasse 7, Brunswick, 38124 Germany; 5 Clinic for Cattle, University of Veterinary Medicine Hanover Foundation, Bischofsholer Damm 15, Hanover, 30173 Germany; 6 Research Institute for Farm Animal Biology, Genome Biology, Wilhelm-Stahl-Allee 2, Dummerstorf, 18196 Germany; 7 Agricultural and Environmental Faculty, University Rostock, Justus-Von-Liebig-Weg 6, Rostock, 18059 Germany; 8 Friedrich-Loeffler-Institut, Federal Research Institute for Animal Health, Südufer 10, Greifswald-Insel Riems, 17493 Germany; 9 Institute for Immunology, University of Veterinary Medicine Hanover Foundation, Bünteweg 2, Hanover, 30559 Germany

**Keywords:** Cattle, Intramammary infection model, Milk parameters, Vaginal temperature, Mastitis, *S. aureus*, *E. coli*, Bayesian model

## Abstract

**Background:**

In dairy cattle, mastitis causes high financial losses and impairs animal well-being. Genetic selection is used to breed cows with reduced mastitis susceptibility. Techniques such as milk cell flow cytometry may improve early mastitis diagnosis. In a highly standardized in vivo infection model, 36 half-sib cows were selected for divergent paternal *Bos taurus* chromosome 18 haplotypes (Q *vs*. q) and challenged with *Escherichia coli* for 24 h or *Staphylococcus aureus* for 96 h, after which the samples were analyzed at 12 h intervals. Vaginal temperature (VT) was recorded every three minutes. The objective of this study was to compare the differential milk cell count (DMCC), milk parameters (fat %, protein %, lactose %, pH) and VT between favorable (Q) and unfavorable (q) haplotype cows using Bayesian models to evaluate their potential as improved early indicators of differential susceptibility to mastitis.

**Results:**

After *S. aureus* challenge, compared to the Q half-sibship cows, the milk of the q cows exhibited higher PMN levels according to the DMCC (24 h, *p* < 0.001), a higher SCC (24 h, *p* < 0.01 and 36 h, *p* < 0.05), large cells (24 h, *p* < 0.05) and more dead (36 h, *p* < 0.001) and live cells (24 h, *p* < 0.01). The protein % was greater in Q milk than in q milk at 0 h (*p* = 0.025). In the *S. aureus* group, Q cows had a greater protein % (60 h*, p* = 0.048) and fat % (84 h, *p* = 0.022) than q cows. Initially, the greater VT of *S. aureus*-challenged q cows (0 and 12–24 h, *p* < 0.05) reversed to a lower VT in q cows than in Q cows (48–60 h, *p* < 0.05).

Additionally, the following findings emphasized the validity of the model: in the *S. aureus* group all DMCC subpopulations (24 h-96 h, *p* < 0.001) and in the *E. coli* group nearly all DMCC subpopulations (12 h-24 h, *p* < 0.001) were higher in challenged quarters than in unchallenged quarters. The lactose % was lower in the milk samples of *E. coli*-challenged quarters than in those of *S. aureus*-challenged quarters (24 h, *p* < 0.001). Between 12 and 18 h, the VT was greater in cows challenged with *E. coli* than in those challenged with *S. aureus* (3-h interval approach, *p* < 0.001).

**Conclusion:**

This in vivo infection model confirmed specific differences between Q and q cows with respect to the DMCC, milk component analysis results and VT results after *S. aureus* inoculation but not after *E. coli* challenge. However, compared with conventional milk cell analysis monitoring, e.g., the global SCC, the DMCC analysis did not provide refined phenotyping of the pathogen response.

**Supplementary Information:**

The online version contains supplementary material available at 10.1186/s12917-024-03996-y.

## Background

Bovine mastitis is one of the most common diseases in dairy cows worldwide [1–3]. In addition to detrimentally affecting animal well-being, it strongly influences dairy farm profitability by direct and indirect effects on milk yield and quality [4, 5]. Intramammary infection (IMI) occurs in 20–50% of all lactating cows [3, 6]. Due to increasing antibiotic resistance, measures to reduce the use of antibiotics to treat mastitis are needed. This includes prevention strategies, including milking hygiene procedures, early mastitis diagnosis and mastitis treatment, supportive treatment of clinical cases, separation of infected cows and culling to reduce IMI at the herd level [1].

In addition, breeding for increased mastitis resistance is essential. Parameters based on phenotypic characteristics such as the somatic cell count (SCC) and milk quality analysis are often used to assess the genetic value of mastitis resistance in dairy cows. In addition to the use of surrogate parameters, new tools for genetic selection, including genomic information, have become state-of-the-art. In earlier studies [7, 8], quantitative trait loci (QTLs) were identified to provide more information about genetically determined molecular mechanisms of mastitis resistance in mammary epithelial cells from cows harboring alternative QTL alleles [9]. These observations were based on previous studies reporting a QTL on *Bos taurus* chromosome 18 (BTA18), which was related to the SCC [10, 11]. Several follow-up studies have confirmed that this major locus segregates in the Holstein dairy cattle population and has a substantial effect on longevity [7, 8]. However, to date, no distinction has been made between the different pathogenetic and pathophysiological backgrounds of mastitis when selecting for mastitis resistance.

*Staphylococcus aureus (S. aureus)* and *Escherichia coli (E. coli)* are two of the most important mastitis pathogens with different clinical disease courses [12, 13]*.* IMI caused by *S. aureus* usually results in subclinical mastitis, which is often less severe but can persist for a lifetime. This chronic course has long-term effects on total milk yield, milk quality, milk composition and overall productivity [4, 14]. Due to the persistence and intermittent shedding of *S. aureus*, detection is difficult [1, 15]. In contrast, *E. coli* causes acute mastitis with moderate to severe clinical signs that can be overcome within a few days but often require veterinary treatment. Severe disease may gravely affect the animal [16]. Because of their opposite clinical outcomes, *S. aureus* and *E. coli* have widely been used in experimental studies for subclinical and clinical mastitis, and these studies have revealed the different underlying mastitis pathophysiologies of *S. aureus* and *E. coli* mastitis [12, 14, 17].

In general, the somatic cell count (SCC) in milk is > 97% leukocytes and < 3% mammary epithelial cells (MECs) [18, 19]. The innate immune response during IMI leads to enhanced recruitment of immune cells, which are important for defense against invading pathogens. The identification of subclinical mastitis is widely based on SCC values > 100,000 cells / ml. However, healthy cows may display higher values [20, 21]. Moreover, inflammation of the mammary gland has even been observed in cows with SCC values below 100,000 cells / ml [22]. Furthermore, the SCC varies with lactation status, age, stress, milking frequency and season [23, 24].

For mastitis control programs, a large variety of diagnostic methods are available for milk sample analysis, including the SCC and the California Mastitis Test (CMT) [25]. The SCC is a widespread tool used to estimate cell quantities for mastitis detection but does not determine the distribution of different cell types.

To further distinguish between cell types in milk, the differential milk cell count (DMCC) has gained increasing attention in recent years [18]. Twenty-five years ago, the DMCC was described to allow the detection of mastitis in its initial phase via the analysis of different immune cell populations in milk [26]. This technique allows for the differentiation of polymorphonuclear neutrophils (PMNs), lymphocytes and macrophages [27] by either microscopic or flow cytometric analysis [19, 20, 22, 28–30].

PMNs constitute more than 90% of the chemokine-attracted migrating leukocyte populations in the alveolar lumen [31]. Alongside resident mammary epithelial cells, these phagocytes serve as one of the first lines of defense against invading pathogens. PMNs are recruited by chemokines secreted from MECs [32]. The maximum PMN influx (3122 ± 458 PMN /µL) in quarters that were inoculated with *E.* *coli* occurred 6–24 h after challenge [33]. In contrast, in cases of chronic mastitis, PMN fractions may vary from very high values, as observed in acute mastitis (~ 56 – 73% of the SCC), to very low percentages, as observed in uninfected quarters (~ 28%). The predominant cell population in the milk of healthy cows is reported to be lymphocytes (~ 47% of the SCC). Their fraction and number seem to be genetically determined [14, 22]. Dosogne et al. reported that lymphocytes and monocytes were more abundant in early lactation than in mid- and late lactation, whereas macrophages and PMNs remained considerably less abundant [34].

Degen et al. assessed the effectiveness of the DMCC as a tool for early mastitis detection based on changes in the relative cell populations for differentiation between healthy and inflamed quarters as well as between acute and chronic mastitis [35]. In summary, the DMCC is advantageous because it can be used to investigate the proportions of different somatic cell populations in milk samples to optimize mastitis diagnosis and to assess the general health of dairy cows. The DMCC is more accurate than the SCC and provides additional information for detecting mastitis in different and early stages as well as for detecting subclinical mastitis in patients with an SCC of < 100,000 cells / ml. The accuracy of the DMCC for detecting subclinical mastitis under field conditions has been evaluated [36], and the DMCC has been suggested as a tool for monitoring disease progression or treatment success [18]. Thus, the DMCC can serve as a tool for preventive health management in dairy cows, allowing early and accurate detection of mastitis.

The aim of the present study was to explore whether cows with similar overall genetic backgrounds (paternal half-sibs) but divergent BTA18 paternal haplotypes show differential early responses to mastitis pathogen challenge in acute or chronic mastitis models. In particular, we hypothesized that compared with conventional parameters, the differential milk cell count (DMCC), milk parameters and vaginal temperature would be better early indicators of a genetically driven differential early response to mastitis pathogen challenge in an acute or chronic mastitis model.

## Results

### Differences in the DMCC

#### Differences in the DMCC between challenged quarters and unchallenged quarters

The DMCC of untreated quarters and quarters treated with saline solution (0.9%) differed significantly in both pathogen groups. In the *E. coli* group at timepoint 24 h *post inoculation* (*p.* *i.*) numbers of cells in all leukocyte subpopulations (*p* < 0.001) were higher in the untreated quarters compared to the quarters treated with saline solution. In the *S. aureus* group from timepoint 24 *p.* *i. *until the end of the experiment PMNs, live cells and SCC were significantly lower in untreated quarters compared to quarters treated with saline solution (p < 0.05, except for 72 h: PMNs p = 0.06, live cells: p = 0.07 and SCC: p = 0.08). Furthermore, from timepoint 36 h p. i. until the end of the experiment, lymphoid cells, large cells and dead cells were significantly lower in untreated quarters compared to quarters treated with saline solution in the *S. aureus* group (p < 0.05, except for 72 h: lymphoid cells and large cells p = n. s., and 72 h and 96 h dead cells: p = n.s.).

No significant differences in the DMCC were found between challenged quarters and untreated or saline solution treated quarters at timepoint 0 h for cows challenged with *E. coli* and at timepoint 0 h and 12 h for cows challenged with *S. aureus,* irrespective of the haplotype (Additional Fig. 1).

At timepoint 12 h and 24 h *p.* *i.* significantly higher cell counts of all cell populations were detected in the *E. coli*-challenged quarters compared to untreated or saline solution treated quarters (*p* < 0.01,). Similarly, *S.* *aureus*-challenged quarters showed significantly higher numbers of cells in all cell populations starting 24 h *p. i.* until the end of the experiment (*p* < 0.001) compared to untreated or saline solution treated quarters (Additional Fig. 1).

#### Differences in the DMCC between cows with haplotypes q vs. Q

**Fig. 1:**
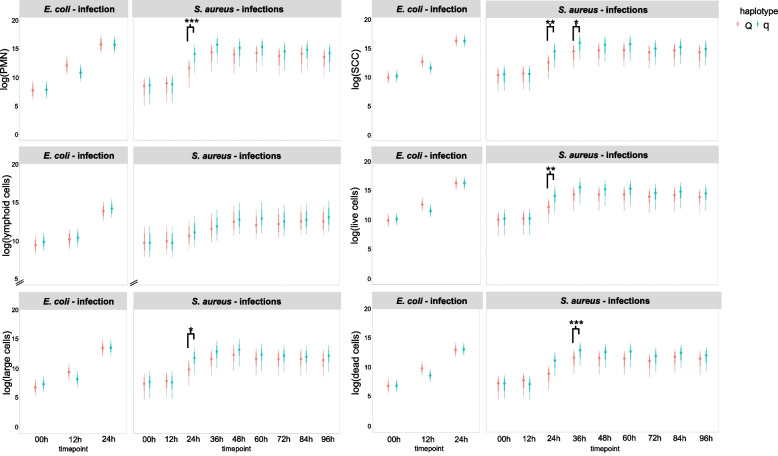
DMCC of milk samples from cows with divergent haplotypes during challenge with *E. coli* or *S. aureus*. Illustration of the Bayesian model including the logarithmized number [cells/ml] of polymorphonuclear neutrophils (PMNs), somatic cell count (SCC), lymphoid cells, large cells, and live and dead cells in the milk of challenged quarters of primiparous cows with either favorable (Q) or unfavorable (q) haplotypes that were challenged with *Escherichia coli (E. coli)* for 24 h or with *Staphylococcus aureus (S. aureus)* for 96 h. The dataset includes *n* = 35 cows, distributed as follows: *E. coli* challenge Q: *n* = 5, q: *n* = 6 and *S. aureus* challenge Q: *n* = 12, q: *n* = 12. Model predictions are presented as 80% and 95% confidence intervals of the mean. Differences between Q and q cows are indicated with * if *p* < 0.05 and with ** if *p* < 0.01 and *** if *p* < 0.001. Significant differences between the pathogen groups (*E. coli vs. S. aureus*) as well as differences over time relative to challenge are not shown

The DMCC of the challenged quarters did not differ significantly between the haplotypes q and Q in the first 24 h *p.* *i*. (Bayesian model independent of the inoculated pathogen, *p* > 0.1, data not shown). Differences between q and Q cows were detected in the *S. aureus*-challenged quarters at later timepoints after inoculation. PMN levels were significantly higher in the challenged quarters of q cows compared to challenged quarters of Q cows at 24 h *p.* *i.* (*p* < 0.001, Fig. 1). Similarly, the SCC at timepoints 24 h *p.* *i*. (*p* < 0.01) and 36 h *p.* *i*. (*p* < 0.05), as well as live cell count (*p* < 0.01) and large cell count (*p* < 0.05) at timepoint 24 h *p.* *i*. and dead cell count at 36 h *p. i.* (*p* < 0.001) were higher in the challenged quarters of q cows compared to challenged quarters of Q cows (Fig. 1).

#### Differences in the DMCC between quarters challenged with *E. coli* and quarters challenged with *S. aureus* within the haplotype groups

There were no significant differences in the DMCC between the *E. coli-* and *S. aureus*-challenged quarters at timepoint 0 h. Within the Q group, at timepoint 12 h *p.* *i.* numbers of all measured cell populations in *E. coli*-challenged quarters were significantly higher than in *S. aureus*-challenged quarters (*p* < 0.05). This cannot be reported for q cows except for PMNs, that were significant higher at timepoint 12 h *p.* *i.* in *E. coli*-challenged quarters compared to *S. aureus*-challenged quarters (*p* = 0.015) within the q group. Except for PMNs within haplotype group q (*p* = 0.061), at 24 h *p.* *i.,* all cell populations were significantly higher in the *E.* *coli-*challenged quarters compared to the *S.* *aureus-*challenged quarters, independent of the cow haplotype (*p* < 0.05) (Fig. 1, only significant differences between haplotype groups, but not between pathogen groups are indicated with asterisks).

### Differences in milk component analysis

#### Differences in milk component analysis between cows with haplotypes q vs. Q

**Fig. 2:**
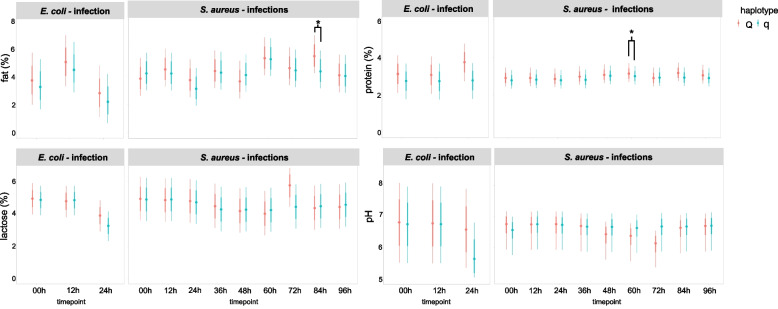
Milk component analysis of milk samples from cows with divergent haplotypes during pathogen challenge. Illustration of the Bayesian model including fat %, protein %, lactose % and pH in milk of all quarters of primiparous cows with either favorable (Q) or unfavorable (q) haplotype, challenged with *Escherichia coli (E. coli)* for 24 h or with *Staphylococcus aureus (S. aureus)* for 96 h. The dataset includes *n* = 35 cows, distributed as follows: *E. coli* challenge Q: *n* = 5, q: *n* = 6 and *S. aureus* challenge Q: *n* = 12, q: *n* = 12. Model predictions are presented as 80% and 95% confidence intervals of the mean. Differences between Q and q cows are indicated with * if *p* < 0.05 and with ** if *p* < 0.01 and *** if *p* < 0.001. Significant differences between the pathogen groups (*E. coli vs. S. aureus*) as well as differences over time relative to challenge are not shown

The overall analysis of all cows and all quarters from both pathogen groups in one Bayesian model revealed significant differences in protein % at 0 h, which was significantly greater in Q than in q cows (*p* = 0.025; Q: mean ± sd = 2.97 ± 0.24; q: median = 2.76, IQR = 0.27; data not shown). Regarding the *S.* *aureus* group, there were significant differences between Q and q cows at later timepoints: the protein % was significantly greater in Q cows at 60 h *p.* *i.* (*p* = 0.048, Fig. 2), as was the fat % at 84 h *p. i.* (*p* = 0.022, Fig. 2). For pH, a trend at 72 h *p.* *i.* was detected to be greater for q than for Q cows (*p* = 0.055). In both pathogen groups, no significant differences were found for the lactose % in quarters of cows with different haplotypes (Fig. 2).

#### Differences in milk component analysis between cows challenged with *E. coli* and cows with *S. aureus*

Concerning the milk component analysis, few differences were found between the applied pathogens. The fat % was significantly greater in *S. aureus*-challenged cows than in *E. coli*-challenged cows at 0 h (*p* < 0.01) and 24 h (*p* < 0.05) (Fig. 2). Similarly, the lactose % was significantly lower in *E. coli-*challenged cows at 24 h *p.* *i.* (*p* < 0.001) than in *S. aureus-*challenged cows. No significant differences in pH or protein % were detected between the two pathogens (Fig. 2, only significant differences between haplotype groups, but not between pathogen groups are indicated with asterisks).

### Differences in vaginal temperature

#### Course of VT during intramammary challenge

**Fig. 3:**
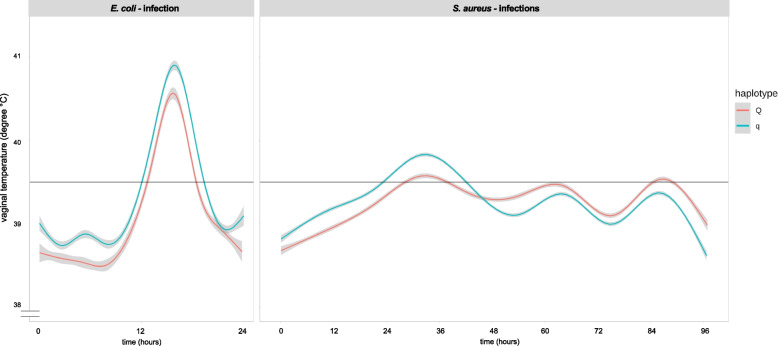
Vaginal temperature of divergent haplotype cows during pathogen challenge. Vaginal temperature (in degrees Celsius) of primiparous cows with either favorable (Q) or unfavorable (q) haplotypes challenged with *Escherichia coli (E. coli)* for 24 h or with *Staphylococcus aureus (S. aureus)* for 96 h. Vaginal temperature was measured every 3 min. The dataset included *n* = 34 cows, distributed as follows: *E. coli* challenge Q: *n* = 5, q: *n* = 5 and *S. aureus* challenge Q: *n* = 12, q: *n* = 12. The ggplot2 package [37] with the geom_smooth function, which uses a generalized additive model (gam), was used for visualization. The gray areas above and below the line represent the 95% confidence intervals, and the intercept is set at 39.5 °C

The analysis of the vaginal temperature (VT), which was measured every three minutes, revealed divergent curves for the two pathogens and between the two different haplotypes, as shown in Fig. 3. The VT of *E. coli-*challenged cows was greater during the whole experiment in q than in Q cows. Vice versa, *S.* *aureus*-challenged cows with haplotype q showed an initially greater VT; however, this reversed after 48 h to a lower VT in q cows than in Q cows. While *E. coli-*challenged cows developed one vaginal temperature peak within the 24 h challenge, *S.* *aureus*-challenged cows showed undulant vaginal temperature during the 96 h challenge. The circadian rhythm of the physiological body temperature during *S. aureus* challenge was affected for at least the first 36 h after infection (Fig. 3).

These findings revealed the effect of intramammary challenge on VT. To uncover significant differences between the pathogen and haplotype groups, the data were further analyzed at 3-h and 12-h intervals.

#### Three-hour interval approach

**Fig. 4:**
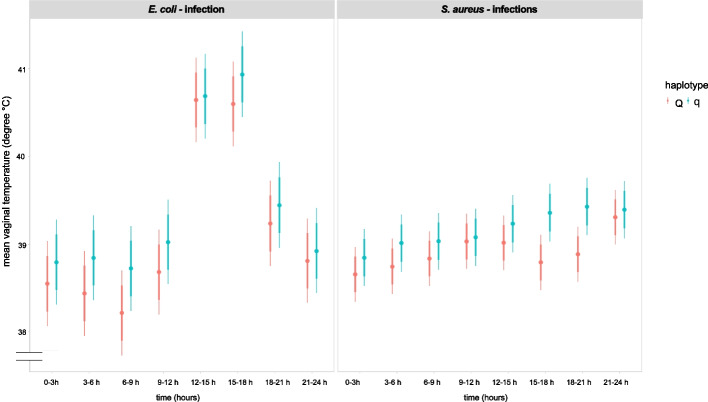
Vaginal temperature of divergent haplotype cows during pathogen challenge illustrated in 3-h intervals. Illustration of the Bayesian model including the vaginal temperature (in degrees Celsius) of primiparous cows with either favorable (Q) or unfavorable (q) haplotypes challenged with *Escherichia coli (E. coli)* for 24 h or with *Staphylococcus aureus (S. aureus)* for 96 h. Vaginal temperature was measured every 3 min and summed into 3-h intervals. The dataset included *n* = 34 cows, distributed as follows: *E. coli* challenge Q: *n* = 5, q: *n* = 5 and *S. aureus* challenge Q: *n* = 12, q: *n* = 12. Model predictions are presented as 80% and 95% confidence intervals of the mean. Statistical analysis did not reveal any significant differences (*p* < 0.05) between Q and q cows. Significant differences between the pathogen groups (*E. coli vs. S. aureus*) as well as differences over time relative to challenge are not shown

With the Bayesian model, when analyzing the vaginal temperature at 3-h intervals, highly significant differences between both pathogens were detected at 12–15 h and 15–18 h after the start of the intramammary challenge. The vaginal temperature was greater in cows challenged with *E. coli* than in those challenged with *S. aureus* (*p*< 0.001; Fig. 4).

No significant differences were found between the two haplotypes (Q versus q) in this 3-h interval analysis (Fig. 4).

#### Twelve-hour interval approach

**Fig. 5:**
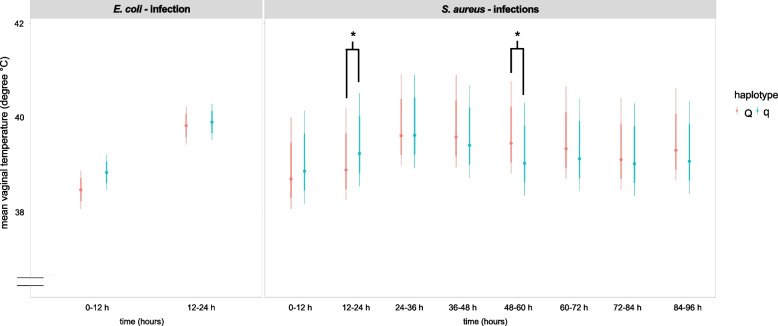
Vaginal temperature of divergent haplotype cows during pathogen challenge illustrated in 12-h intervals. Illustration of the Bayesian model including the vaginal temperature (in degrees Celsius) of primiparous cows with either favorable (Q) or unfavorable (q) haplotypes challenged with *Escherichia coli (E. coli)* for 24 h or with *Staphylococcus aureus (S. aureus)* for 96 h. The vaginal temperature was measured every 3 min and summarized into 12-h intervals. The dataset included data for 34 cows, distributed as follows: *E. coli* challenge Q: *n* = 5, q: *n* = 5 and *S. aureus* challenge Q: *n* = 12, q: *n* = 12. Model predictions are presented as 80% and 95% confidence intervals of the mean. Differences between Q and q cows are indicated with * if *p* < 0.05 and with ** if *p* < 0.01 and *** if *p* < 0.001. Significant differences between the pathogen groups (*E. coli vs. S. aureus*) as well as differences over time relative to challenge are not shown

Using a 12-h interval approach, a comparison between the haplotypes revealed significant differences between Q and q cows. The two haplotypes did not differ in the *E. coli*-challenged group (*p* > 0.1, Fig. 5), but in the *S. aureus* group in the interval 12–24 h *p. i.* (*p* = 0.029), the vaginal temperature was significantly greater in q than in Q cows. Vice versa, in the interval of 48–60 h *p. i.* (*p* = 0.011), the vaginal temperature was significantly lower in q cows than in Q cows (Fig. 5).

## Discussion

The aim of the present study was to compare the differential milk cell count (DMCC), parameters of milk component analysis and vaginal temperature between two divergent paternal haplotype groups (Q *vs*. q) during a controlled intramammary challenge experiment with either *E. coli* or *S. aureus* using Bayesian models. In our study, we started with the hypothesis that compared to conventional parameters, the DMCC, milk parameters and vaginal temperature would be better early indicators of a genetically driven differential early response to a mastitis pathogen challenge in an acute or a chronic mastitis model.

As expected, leukocyte subpopulations differed significantly between challenged and unchallenged quarters. This was the case for SCC, PMNs and live cell counts, that were significantly greater in the milk of the challenged quarters than in that of the unchallenged quarters 24 h after inoculation of *S. aureus*. In contrast, in the *E. coli* group all cell populations, except for lymphoid cells, were significantly higher in challenged compared to unchallenged quarters already 12 h after inoculation. This finding is in line with observations reported in several other studies [12, 13].

Although the differences were less pronounced than expected, we confirmed our hypothesis regarding DMCC differences between Q and q cows in the *S. aureus* model: the DMCC showed significantly more PMNs in the milk of q cows than in that of Q cows at 24 h, a greater SCC at 24 and 36 h, an elevated number of live and large cells (24 h *p.* *i.)* as well as dead cells (36 h *p.* *i.)* after challenge. Such differences were not observed in the acute mastitis model, as no differences between Q and q cows were detected after intramammary infection with *E. coli*. This might be due to the experimental setup with a shorter period of observation after inoculation with *E. coli* (24 h) than after inoculation with *S. aureus* (96 h), which is a limitation of the present study. Another limitation of this experimental setup of this study involves the comparability of untreated udder quarters und quarters treated with saline solution. Surprisingly, DMCC in milk samples of these quarters differed significantly in the *E. coli* group (12 h and 24 h) as well as in the *S. aureus* group (24 h - 96 h). This was unexpected, as these two treatments were both meant to serve as negative controls. The observed differences cannot be explained by the fact, that on one treatment group saline solution was inserted and in the other treatment group the teat channel and udder parenchyma was not touched, because both pathogen groups showed inverse results. In the *S. aureus* group, the untreated quarters were lower in cell counts compared to the saline solution treated quarters (numerically at 0 h and 12 h, significantly 24 h - 96 h). But in the *E. coli* group it was the other way around: untreated quarters showed higher numbers of leukocyte subpopulations compared to quarters treated with saline solution. It can be speculated that these differences originate from the allocation of the treatment groups, which were different in the two pathogen groups: In the *E. coli* group the hind left quarter was treated with saline solution, and the front right and front left quarters remained untreated. In the *S. aureus* group, the front left quarter was treated with saline solution, and the front right quarter remained untreated. These differences in DMCC between untreated quarters and saline solution were relatively low and had no impact on clinical parameters as swelling or pain. However, this aspect must be taken into account in future study designs.

Overall, the findings concerning the dynamics of the different cell populations in the milk of challenged quarters are in accordance with a study from Rivas et al. (2001), who found PMNs to be the predominant subpopulation during an intramammary challenge experiment [20]. In the same study, the authors demonstrated the possibility of detecting nonmastitic, early inflammatory and late inflammatory reactions within their study population (inoculated *S. aureus* in six lactating cows) via DMCC analysis. They demonstrated an early increase in the percentage of PMNs and a decrease in lymphocytes one day after inoculation. At days four to eight, PMNs were the predominant subpopulation, but the percentage of mononuclear cells increased [20]. Using the DMCC, Wall et al. observed a shift in different cell populations, predominantly PMNs, even at low SCC levels after intramammary treatment with LPS [27]. A study from Damm et al. revealed increasing proportions of PMNs and decreasing proportions of macrophages as the SCC increased, whereas the lymphocyte population remained consistently lower than the other cell populations [30].

The cows included in this study were genetically selected via SNP genotyping for alternative paternal chromosome 18 haplotypes associated with favorable (Q) or unfavorable (q) effects on the health of the mammary gland. During the selection procedure, the somatic cell score (SCS) served as a surrogate for mastitis incidence [10, 38–40]. Beyond that, strict selection criteria, for example, only primiparous cows, equal housing conditions, and a highly standardized experimental setup and data collection, were applied to reach the maximal scientific output. Furthermore, Bayesian models including variable and fixed effects were used as up-to-date and robust statistical analyses to complete the high-grade data analysis. The consideration of individual cow effects was applied in the Bayesian models to achieve the most accurate predictions.

To date, other in vivo studies that investigate DMCC in the milk of cows with divergent genetics regarding mastitis resistance in general and with respect to the target region on BTA18 in particular are lacking. Although the confirmed quantitative trait locus (QTL) on BTA18 has been associated with important performance traits, such as calving ease and stillbirth [41–44], several authors have attempted to identify the exact causal mutation, which has recently been further investigated by Dachs et al. [45].

In parallel to the in vivo infection model, a long-term trial including *n* = 6 selected cows (Q: *n* = 3, q: *n* = 3) for two lactation periods was performed by our scientific group at the Research Institute for Farm Animal Biology in Dummerstorf, Germany. In the long-term trial, it was shown that the average SCC was significantly greater in q cows than in Q cows [46], and these individuals had more subclinical mastitis. This finding is in line with a greater PMN level and SCC after pathogen challenge in the q cows in the present study. Additionally, in a previous publication of this group including the same set of cows used for the intramammary infection model, quarter milk yield and bacterial shedding were analyzed. No significant differences between the haplotypes regarding quarter milk yield could be detected, but the total milk yield decrease 12 h and 24 h after the start of the challenge was minor in Q cows compared to q cows. Concerning the bacterial shedding in milk, cows with haplotype Q showed fewer colony-forming units in milk samples from challenged quarters 12 h after intramammary inoculation than did those with haplotype q [38].

In addition to the DMCC analysis, within this study, the milk parameters protein %, fat %, lactose % and pH were analyzed at the quarter milk level during the challenge experiment. These serve as the most important parameters for describing milk composition and can be interpreted in the context of important physiological processes such as energy balance and changes in the blood–milk barrier. In the case of IMI, pathogen-dependent changes in milk yield and quality have been reported [47, 48]. In addition to negative correlations between the SCC and milk yield, positive correlations between the SCC and the percentage of fat and protein were also reported [49]. Conversely, negative correlations were found between IMI and the milk parameters fat, protein and lactose [50]. Clinical signs of mastitis coincided with lower lactose and higher protein concentrations. In the same clinical trial, greater changes in milk yield were observed for IMI with *E. coli* than with *S. aureus*, but *S. aureus* IMI led to slightly reduced lactose concentrations [47]. Similarly, in other studies that included mastitis-causing pathogens such as *S. aureus*, reduced lactose was found in patients with subclinical mastitis, but no difference in protein or fat content was reported [51]. In the present study, at 24 h, the lactose % was significantly lower in the milk of *E. coli*-challenged cows than in that of *S. aureus*-challenged cows, which can be explained by the reduced synthesis capacity of MECs due to the inflammatory response. In addition, IMI changes blood-milk barrier permeability, which leads to a concurrent efflux of lactose and potassium into the bloodstream and a simultaneous increase in sodium, chloride and proteins from the bloodstream into the milk [48]. Because lactose is exclusively produced in the mammary gland and cannot pass through an intact epithelial barrier [52], this difference in lactose may also be explained by leakage through the impaired tight junctions of the MEC [53]. Concerning the comparison of pathogen-specific effects, we most likely detected a lower lactose % in the *E. coli* group, as inflammation was more vigorous and bacterial shedding was greater [38], indicating greater metabolism of lactose and, therefore, a lower lactose % in the *E. coli* group than in the *S. aureus*-challenged group. Furthermore, the severity of clinical signs during mastitis coincides with lower lactose concentrations in cows with IMI caused by *S. aureus, Streptococcus uberis* and *E. coli*, with the greatest decrease in *E. coli*-affected animals [47]. However, as the setup of the present studies does not allow for mechanistic conclusions, these explanations remain speculative.

Interestingly, the protein % was significantly greater in milk from Q cows than in milk from q cows at 0 h. Regarding the *S. aureus* group, Q cows had a significantly greater protein % in milk at 60 h. Although these differences might be side effects, it is noteworthy that in both pathogen groups at all other timepoints, the protein % was also numerically greater in Q cows than in q cows.

As anticipated, we also showed that the vaginal temperature was significantly greater in *E. coli*-challenged cows than in *S. aureus-*challenged cows between 12 and 18 h *p.* *i.* This finding is in line with previous studies demonstrating a greater and faster increase in rectal body temperature in *E. coli*-challenged cows [13]. However, in the present study, we detected small-scale changes throughout the entire experimental period due to the use of intravaginal temperature loggers. The measurement of vaginal temperature with loggers has the advantage of detecting diurnal and accurate changes in body temperature compared to measuring rectal body temperature with thermometers [54]. An increase in body temperature is an important reaction of the host to fight against invading pathogens. As reported by several authors, MECs are more capable of inducing stronger cytokine and chemokine synthesis after IMI with *E. coli* than after IMI with *S. aureus* and are responsible for the activation of immune functions to eradicate pathogens [55–57]. This also explains the faster and greater increase in body temperature in *E. coli*-inoculated cows, as shown in our study.

In addition, our study revealed haplotype-dependent differences concerning vaginal temperature. In the *E.* *coli*-challenged group, Q and q cows showed comparable reactions, with a constant increase in vaginal temperature with the onset of inflammation. In contrast, the initial increase in the vaginal temperature of *S. aureus*-challenged q cows at 0 h and 12–24 h reversed to a decrease in the vaginal temperature in q cows compared to Q cows at 48–60 h. As previously published, our group showed that the incidence of metritis after calving was lower in Q cows than in q cows and that Q cows rarely developed fever [40, 46]. These findings suggest that Q cows are less susceptible to infectious diseases than q cows are. Examining the (patho)physiological mechanisms underlying these differences requires further study.

Taken together, these main findings concerning the pathogen comparison are consistent with the existing body of literature and emphasize the validity of this intramammary challenge model. The use of two pathogens, *E. coli* and *S. aureus*, to model acute and chronic mastitis is widely acknowledged, as previously reviewed by Petzl et al. [17]. However, to the best of our knowledge, this is the first IMI model study to compare two divergent BTA18 haplotypes (favorable Q *vs*. unfavorable q) during a controlled intramammary challenge with either *E. coli* or *S. aureus.*

## Conclusion

In conclusion, specific differences in the DMCC, milk parameters and vaginal temperature could be detected between diverging haplotype cows after intramammary infection with *S. aureus*. However, the potential of the DMCC for the refined identification of early response differences to mastitis pathogens could not be confirmed.

Taken together, this study showed that with a highly standardized intramammary challenge experiment combined with robust statistical analyses, it was possible to observe differences between primiparous cows inheriting divergent paternal haplotypes. The results suggest that haplotype selection for mastitis susceptibility works but is far more complex than expected. These findings are relevant to the field, as they provide more information about the connection between genetics and immune functions, leading to mastitis susceptibility.

## Methods

### In vivo infection model

**Fig. 6:**
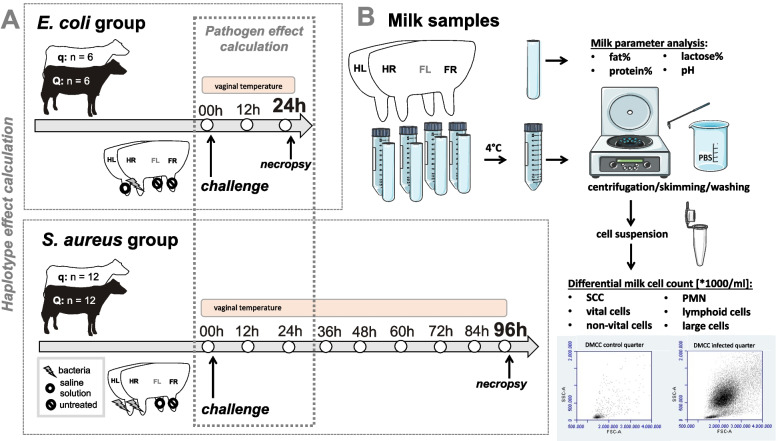
Experimental setup (5A) and sample processing (5B) of the in vivo infection model. **A** Illustration of the experimental setup including *n* = 36 primiparous Holstein Friesian cows with either favorable (Q) or unfavorable (q) BTA haplotypes. After intramammary challenge with *Escherichia coli (E. coli)* for 24 h, the hind right (HR) quarter was challenged, the hind left (HL) quarter was treated with saline solution as a control quarter, and the front right (FR) and front left (FL) quarters remained untreated. After challenge with *Staphylococcus aureus (S. aureus)* for 96 h, the HR and HL quarters were challenged, the FL was treated with saline solution, and the FR remained untreated. Cows were milked with a quarter milking system every 12 h during the challenge to assess full milk samples and milk yield at the quarter level, and vaginal temperature was measured every 3 min during the challenge. **B** Illustration of milk sample processing for milk component analysis and differential milk cell count (DMCC). Milk samples were stored at 4 °C, and one set of samples per cow at each timepoint was analyzed for the milk parameters fat %, protein %, lactose % and pH. With another set, several washing, skimming and centrifugation steps were performed until the cell suspension was analyzed for the DMCC via flow cytometry to assess the absolute somatic cell counts (SCCs) and numbers of polymorphonuclear neutrophils (PMNs) and live, dead, lymphoid and large cells

In total, 36 cows were included in this study, which was conducted between January and September 2016 at the Clinic for Cattle at the University of Veterinary Medicine Hanover (TiHo). The study was approved by the Lower Saxony Federal State Office for Consumer Protection and Food Safety (reference number 33.12–42,502–04–15/2024; approval date: December 15th, 2015). Previously published results included the observed differences between the favorable (Q) and unfavorable (q) BTA18 haplotype half-sib cows before and after parturition [40] as well as the details concerning the in vivo infection model [38]. In brief, 36 primiparous Holstein Friesian cows were selected based on two divergent paternal BTA18 haplotypes, with a focus on the SCC as a marker for udder health and mastitis susceptibility (favorable haplotype Q *n* = 18, unfavorable haplotype q *n* = 18). The cows were purchased from German conventional private dairy farms. From at least four weeks before the expected calving until the end of the intramammary challenge experiment, the animals were kept in individual pens on straw at the Clinic for Cattle, TiHo Hanover. The cows received component rations adjusted to their lactation/gestation status and milk yield, and they were intensively monitored. Details concerning the selection process, daily management of the cows and clinical examination have previously been published [40]. On Day 36 ± 3 after calving, the cows received an intramammary challenge with either *E. coli*_*1303*_ (Q: *n* = 6, q: *n* = 6) or *S. aureus*_*1027*_ (Q: *n* = 12, q: *n* = 12) followed by necropsy after 24 h in the case of *E. coli* challenge or after 96 h in the case of *S. aureus* challenge (Fig. 6A). We decided to use differing numbers of animals between the two pathogen groups because it was an important goal to use the lowest animal number possible according to the 3R principle but still generate robust results and detect differences between the two haplotypes. The number of animals subjected to both challenges was allocated via a power analysis that respected the increase in the SCC after intramammary challenge with *E. coli* and *S. aureus*, which is the key surrogate for mastitis susceptibility during the Q/q haplotype selection process. As previously published by our group, the increase in the SCC is greater and more homogenous in *E. coli*-challenged cows than in *S. aureus-*challenged cows [13]. Therefore, a smaller number of animals is needed to detect significant differences within the *E. coli* group. The rationale behind the two different time courses was animal welfare. It is known that the course of inflammation after infection with *E. coli* is acute and associated with a strong increase in body temperature and severe clinical signs of inflammation, including swelling and pain, in the affected udder quarter. In contrast, the course of inflammation after infection with *S. aureus* is less severe, less homogenous and less chronic. To avoid excessive suffering of the cows included in the study and to stay within the ranges of cancellation criteria, the duration of the follow-up period after intramammary challenge was shorter in the *E. coli* group than in the *S. aureus* group. For *E. coli* challenge, the right hind quarter was inoculated with the pathogen, whereas the left hind quarter was inoculated with saline solution, and both front quarters served as untreated negative control quarters. In the case of *S. aureus* challenge, both hindquarters were inoculated with the pathogen, whereas the left front quarter was inoculated with saline solution, and the right front quarter served as the untreated negative control quarter (Fig. 6A). Intramammary infection was successfully induced in all cows enrolled in the in vivo infection model. Each cow developed clinical signs of mastitis, and the respective pathogen was repeatedly recovered from the milk samples after the inoculation of every cow. The clinical results of the in vivo infection model have previously been published [38].

### Milk component analysis and the DMCC

**Fig. 7:**
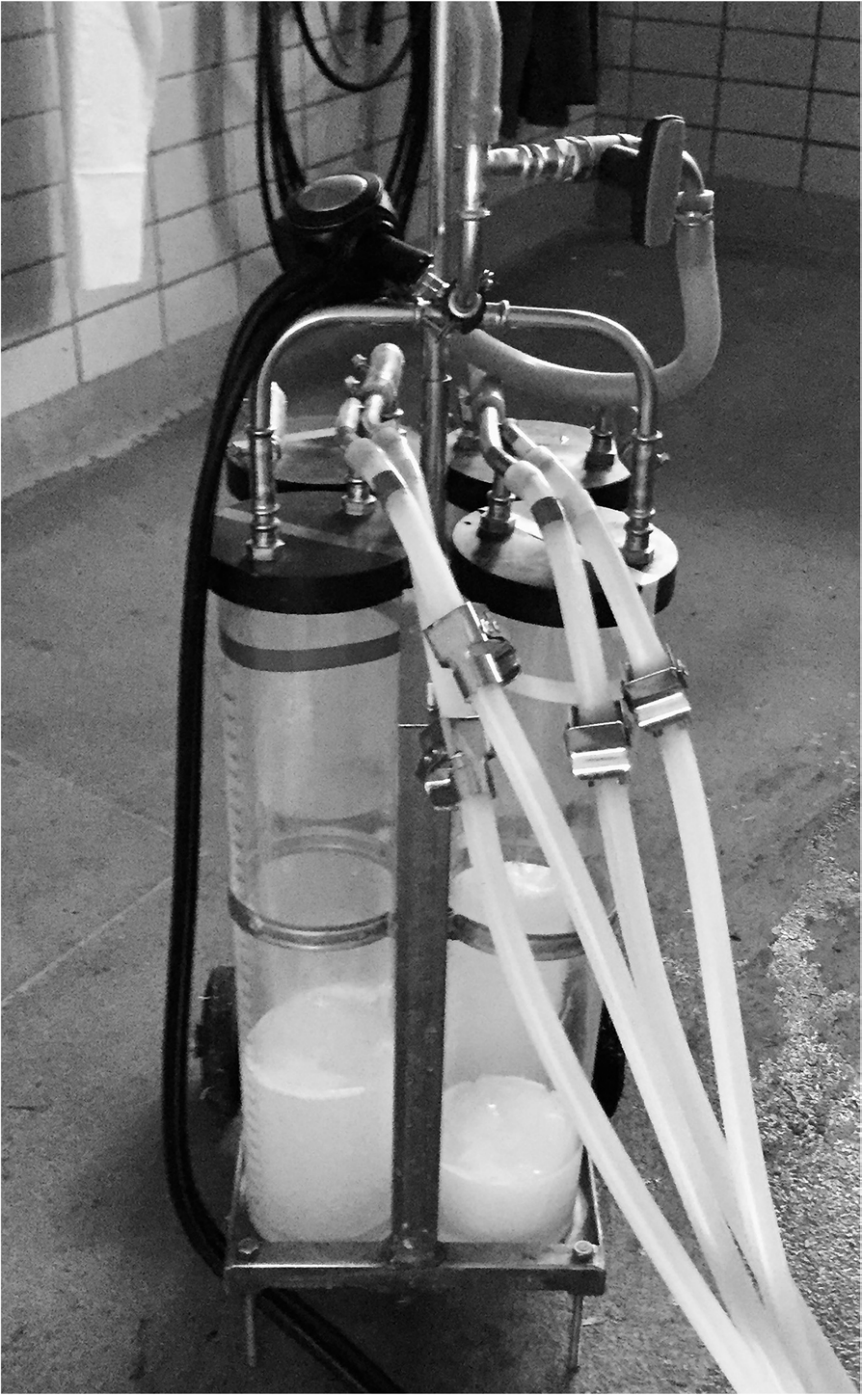
Quarter milking system. Photo of the quarter milking system, which was used every 12 h during the challenge experiment to milk the cows to assess the milk yield and representative full milk samples at the quarter level

During the intramammary challenge experiment, the cows were milked every twelve hours with a quarter milking system (Fig. 7) to assess the milk yield, and representative samples were collected at the quarter level at each timepoint (*E. coli*: 0 h, 12 h, 24 h; *S. aureus*: 0 h, 12 h, 24 h, 36 h, 48 h, 60 h, 72 h, 84 h and 96 h) (Fig. 6A).

The SCC, DMCC and milk parameters were analyzed for each milk sample (Fig. 6B). One full-quarter milk sample (Volume 50 ml) from each cow at each timepoint was preserved with bronopol, stored at 4 °C, transported on dry ice and analyzed via MilkoScanTM FT + (Foss Analytics, Denmark) at Milchwirtschaftlicher Kontrollverband Mittelweser e.V. (Rehburg-Loccum, Germany) for the fat %, protein %, lactose %, pH and SCC.

For flow cytometric analysis, 50 ml per quarter was collected into a centrifuge tube™ (Corning CentriStar, Fisher Scientific GmbH, Schwerte, Germany), placed on ice for transport to the laboratory and stored at 4 °C until further processing to isolate the milk cells for the DMCC (Fig. 6B). The first centrifugation step was performed at 1,000 g and 4 °C for 10 min (Universal 32 R, Hettich, Tuttlingen, Germany), followed by removal of the cream layer and discarding of the supernatant. To suspend the cell pellet, 50 ml of phosphate-buffered saline (PBS, Merck KGaA, Darmstadt, Germany) was added. The second centrifugation was performed at 400 × g and 4 °C for 10 min. The remaining cream was removed, and the final centrifugation step was performed at 250 × g and 4 °C for 10 min. Then, the remaining cell pellet was resuspended in 1 ml of PBS by vortexing (Vortex ZX3, Velp Scientifica, Usmate (MB), Italy) and labeled for flow cytometry. Next, 50 µl of the milk cell suspension was transferred to a 1.5 ml Eppendorf tube (Eppendorf, Wesseling-Berzdorf, Germany). Then, 100 µl of diluted propidium iodide (PO, Sigma‒Aldrich, Taufkirchen, Germany) and 50 µl of diluted acridine orange (AO, 0.01%, Polysciences Inc., Warrington, USA) were added. Both AO and PI are DNS-intercalating dyes, but PI can stain only necrotic cells. Therefore, the combination of AO and OI was used to differentiate between live and dead cells.

Flow cytometric analysis of the milk cell suspension samples was performed with a BD AccuriTM C6 Plus flow cytometer (BD Biosciences, Franklin Lakes, USA). For each sample, 20,000 events were counted and displayed as dot plots with CFlow Sampler Software (BD Biosciences, Franklin Lakes, USA). The cells that were labeled PI positive and AO positive were categorized as dead cells. The cells that were labeled PI negative and AO positive were categorized as live cells. According to previous studies [22, 29, 58], characteristics measured via forward scatter (FSC, size-related) and side scatter area (SSC, complexity- and granularity-related) allow for the differentiation of live cells into lymphoid cells, PMNs and large cells. Gating strategy to determine fractions of leukocytes among milk cells was performed in four steps: (1) Identification of nucleated cells in a PI/SSC-Area density plot after staining with AO. (2) Identification of viable, PI-negative cells in a PI/SSC-Area density plot. (3) Identification of single AO + /PI- cells in an FSC-Area/FSC-Height density plot. (4) Identification of major cell populations among single AO + /PI- milk cells in a FSC-A/SSC-A density plot. PMNs, lymphoid cells, and large cells were identified according to Mehne et al. (2010) [58], who used cell type-specific antibodies. The gating strategy is illustrated in Additional Fig. 2.

It can be assumed that the events recorded as lymphoid cells with this technique include B and T cells as well as natural killer cells of small size. Events recorded as PMNs include neutrophilic granulocytes, whereas events recorded as large cells are macrophages, epithelioid cells, monocytes and large natural killer cells. The percentages of live cells, lymphoid cells, PMNs and large cells were calculated with CFlow Sampler software and transferred to the central project database until further statistical analysis. The percentage of dead cells was calculated as ‘100%-percentage of live cells’. Although the SCC was measured together with the other milk parameters (fat %, protein %, lactose % and pH) via MilkoScanTM FT + analysis (see above), it is mentioned as part of the DMCC in our study because the SCC built the basis for absolute number calculation of the PMNs, live, dead, lymphoid and large cell subgroups. Absolute numbers per ml were calculated by multiplication of the percentages with the SCC per ml.

### Vaginal temperature monitoring

During the whole intramammary challenge experiment, the vaginal temperature was measured every 3 min. Therefore, a vaginal data logger (HOBO U12 Stainless Temperature (4,900 ft.), Onset Computer Corporation, Bourne, Massachusetts/USA) was inserted on a plastic device (EAZI-BEED CIDR-blank, Zoetis, USA) and placed into the vagina of the cow (Fig. 6A) as previously described by Smith et al. and Espejo et al. [59]. The data were recorded on a USB mass storage device. At the end of the experiment, shortly before necropsy, the vaginal temperature logger was removed, and the sample was placed directly into cold water to mark the extraction timepoint. The data were saved from the USB device. For each cow, only the data from the start of the experiment until extraction of the logger were transferred to the central project database until further statistical analysis. All values < 36.5 °C were excluded because they were nonphysiological. In one cow, missing values occurred because the logger had to be reinserted multiple times. Hence, a total of 54.349 data points for *S. aureus*-challenged cows and 4.728 data points for *E. coli*-challenged cows were analyzed. For further analysis, mean values for each cow at 3-h and 12-h intervals were calculated. Both interval approaches were applied to analyze the differences between the two pathogen groups *(E. coli vs. S. aureus)* from 0 h until 24 h (*E.* *coli*-challenge ended) as well as for comparisons between the two BTA18 haplotype groups (Q *vs.* q).

### Statistical analysis

Concerning the data analysis included in this part of the project, one cow (haplotype Q, pathogen *E. coli*) was excluded. This cow had a congenital defect of the teat channel in two udder quarters. The two teat channels were not consistent, so it was only possible to milk this cow on the remaining two functional udder quarters. As the cows were selected for the experiment before their first calving, this defect was not detected until parturition. The cow reached an adequate average daily milk yield and was subjected to *E. coli* challenge. However, concerning the data presented here, we decided to exclude this cow and only use animals with four intact udder quarters. Therefore, the dataset used for the DMCC and milk component analyses included 35 cows, which were subjected to the following conditions: *E. coli* challenge, Q: *n* = 5; q: *n* = 6 for 24 h; and *S. aureus* challenge, Q: *n* = 12, q: *n* = 12 for 96 h. Due to a lack of data transfer, another cow (haplotype q, pathogen *E. coli*) had to be excluded from the vaginal temperature analysis. All the data were refined in Microsoft Excel (Microsoft Corporation, Microsoft Excel, 2018) and analyzed using R statistical software [60]. In the case of missing values (< 1%), data were collected during the analysis in R statistical software using the ‘missRanger’ package [61].

Data analysis of the DMCC and SCC, milk component analysis and vaginal temperature was performed using Bayesian models with the R package ‘brm’ [62, 63]. The response variable was either a DMCC parameter (SCC, PMNs, lymphoid cells, large cells, live cells or dead cells), a milk parameter (fat %, protein %, lactose % or pH) or the mean vaginal temperature during a 3-h or 12-h interval. Cow individuality was considered within the model via random effects on the intercept, and interactions between haplotype and timepoint (haplotype*timepoint) or between the respective pathogen and timepoint (pathogen*timepoint) for repeated measures were also assessed. These models were used to determine differences between challenged and unchallenged quarters (“treatment”: challenged *vs*. untreated *vs*. saline solution), differences between the two pathogens (*E. coli vs*. *S. aureus* during the first 24 h *p. i.*) and differences between the haplotypes (Q *vs*. q) during the first 24 h in *E. coli*-challenged cows and for 96 h in *S. aureus*-challenged cows.

The analysis of the DMCC (data log transformed), milk components and VT with the Bayesian models was performed as as listed in Table 1.

**Table 1:** Bayesian models for analysis of differential milk cell count (DMCC), milk components and vaginal temperature (VT)

***DMCC***
Analysis of all cows and all quarters from both pathogen groups in one Bayesian model:
Model = brm (log(“response variable”) ~ haplotype * pathogen* timepoint + (1|cow))
Differences between challenged and untreated or saline solution treated quarters (irrespective of haplotype):
Model(E. coli) = brm(log(“response variable”)~ treatment * timepoint + (1|cow))
Model(S. aureus) = brm(log(“response variable”)~ treatment * timepoint + (1|cow))
Differences between haplotypes were analyzed using the following code (filtered for treatment, only inoculated quarters):
Model(E. coli) = brm (log(“response variable”) ~ haplotype * timepoint + (1|cow))
Model(S. aureus) = brm (log(“response variable”) ~ haplotype * timepoint + (1|cow))
Differences between pathogens were analyzed using the following code (filtered for treatment, only inoculated quarters):
Model(Q) = brm (log(“response variable”) ~ pathogen * timepoint + (1|cow))
Model(q) = brm (log(“response variable”) ~ pathogen * timepoint + (1|cow))
***Milk component analysis***
Analysis of all cows and all quarters from both pathogen groups in one Bayesian model:
Model = brm (“response variable” ~ haplotype * pathogen* timepoint + (1|cow))
Differences between haplotypes were analyzed using the following (not filtered for any treatment, all quarters are modeled together):
Model(E. coli) = brm (“response variable” ~ haplotype * timepoint + (1|cow))
Model(S. aureus) = brm (“response variable” ~ haplotype * timepoint + (1|cow))
Differences between pathogens were analyzed using the following code (not filtered for any treatment, all quarters are modeled together):
Model(Q) = brm (“response variable” ~ pathogen * timepoint + (1|cow))
Model(q) = brm (“response variable” ~ pathogen * timepoint + (1|cow))
***Vaginal temperature***
Differences between the haplotypes used to analyze the mean vaginal temperature (mean_VT) at both the 3-h and 12-h intervals were analyzed using the following model:
Model(E. coli) = brm(mean_VT ~ haplotype * time_interval + (1|cow))
Model(S. aureus) = brm(mean_VT ~ haplotype * time_interval + (1|cow))
Model(Q) = brm(mean_VT ~ pathogen * time_interval + (1|cow))
Model(q) = brm(mean_VT ~ pathogen * time_interval + (1|cow))

Post hoc pairwise comparisons among groups after fitting a model were performed using the package ‘emmeans’ [64] for estimated marginal means.

To calculate the *p* values for the pairwise comparisons from the Bayesian models, the package ‘bayestestR ‘ was used [65].

To calculate the differences in protein % between Q and q cows at 0 h, the data were tested for a normal distribution and are presented as the mean/median and standard deviation (SD)/interquartile range (IQR).

*P* values between 0.05 and 0.01 were regarded as statistically significant (*). *P* < 0.01 was regarded as significant (**), and *p* < 0.001 was regarded as highly significant (***).

### Supplementary Information

**Additional file 1:** **Additional Figure 1.** DMCC of quarter milk samples of differentially challenged udder quarters. Illustration of the Bayesian model including logarithmized number [cells/ml] of polymorphonuclear neutrophils (PMN), somatic cell count (SCC), lymphoid cells, large cells, vital and non-vital cells in milk of challenged (one/two quarter/s), *versus* non-infected (one quarter), *versus* control quarters (one quarter, treated with saline solution 0.9%) of uniparous cows challenged with *Escherichia coli (E. coli)* in one udder quarter for 24 hours or with *Staphylococcus aureus (S. aureus)* in two udder quarters for 96 hours. The dataset includes *n* = 35 cows, distributed as follows: *E. coli* challenge: *n* = 11 and *S. aureus* challenge: *n* = 24. Model predictions are presented as 80% and 95% confidence intervals of the mean. Differences between non-infected, control and infected quarters are indicated with * if *p* < 0.05 and with ** if *p* < 0.01 and *** if *p* < 0.001. Significant differences between the haplotype groups (Q *vs.* q), pathogen groups (*E. coli vs. S. aureus*) as well as differences over time relative to challenge are not shown.

**Additional file 2: Additional Figure** **2.** Illustration of gating strategy for differential cell count in milk samples. Gating strategy to determine fractions of leukocytes among milk cells. A) Identification of nucleated cells in a PI/SSC-A density plot after staining with acridine orange (AO). B) Identification of viable, propidium iodide (PI)-negative cells in a PI/SSC-A density plot. C) Identification of single AO+/PI- cells in a FSC-area/FSC-height density plot. D) Identification of major cell populations among single AO+/PI- milk cells in a FSC-A/SSC-A density plot. PMN (polymorphonuclear leukocytes), lymphoid cells, and large cells were identified according to Mehne et al. (2010) [58], who used cell type-specific antibodies to identify polymorphonuclear neutrophils (PMN), lymphoid cell subpopulations, monocytes, and macrophages. FSC, forward scatter; SSC, side scatter.

## Data Availability

The primary data and the applied statistical codes used in this study are available from the corresponding author on reasonable request.

## Abbreviations

%: Percent
BTA18: *Bos taurus* Autosome 18
CMT: California Mastitis Test
DMCC: Differential Milk Cell Count
*E. coli*: *Escherichia coli*
e. g.: Exempli gratia
h: Hours
IMI: Intramammary infection
IQR: Inter quartile range
LPS: Lipopolysaccharide
MEC: Mammary epithelial cells
ml: Milliliter
*p. i.*: Post inoculation
PMNs: Polymorphonuclear neutrophils
QTL: Quantitative trait locus
*S. aureus*: *Staphylococcus aureus*
SCC: Somatic cell count
SCS: Somatic Cell Score
SD: Standard deviation
SNP: Single nucleotide polymorphism
TiHo: University of Veterinary Medicine Hanover
USB: Universal series bus
VT: Vaginal temperature
µL: Microliter
