# Supplementary figures and images for: Cows with diverging haplotypes show differences in differential milk cell count, milk parameters and vaginal temperature after S. aureus challenge but not after E. coli challenge
Source: BMC Vet Res. 2024 May 15;20:200. doi: 10.1186/s12917-024-03996-y (PMC11094921; doi:10.1186/s12917-024-03996-y)

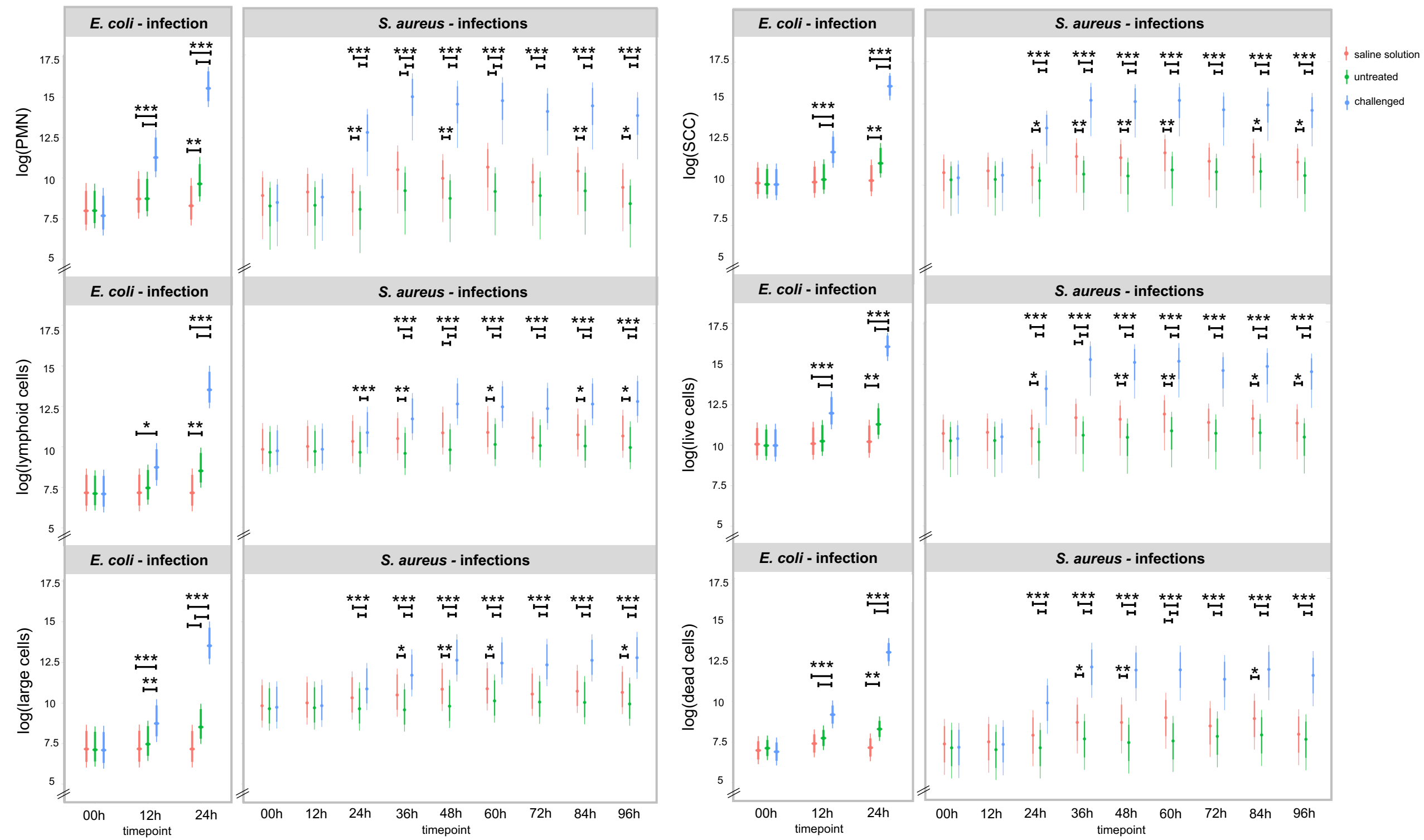

Supplement: Supplementary file 1 — Additional file 1: Additional Figure 1. DMCC of quarter milk samples of differentially challenged udder quarters. Illustration of the Bayesian model including logarithmized number [cells/ml] of polymorphonuclear neutrophils (PMN), somatic cell count (SCC), lymphoid cells, large cells, vital and non-vital cells in milk of challenged (one/two quarter/s), versus non-infected (one quarter), versus control quarters (one quarter, treated with saline solution 0.9%) of uniparous cows challenged with Escherichia coli (E. coli) in one udder quarter for 24 hours or with Staphylococcus aureus (S. aureus) in two udder quarters for 96 hours. The dataset includes n = 35 cows, distributed as follows: E. coli challenge: n = 11 and S. aureus challenge: n = 24. Model predictions are presented as 80% and 95% confidence intervals of the mean. Differences between non-infected, control and infected quarters are indicated with * if p < 0.05 and with ** if p < 0.01 and *** if p < 0.001. Significant differences between the haplotype groups (Q vs. q), pathogen groups (E. coli vs. S. aureus) as well as differences over time relative to challenge are not shown. [file 12917_2024_3996_MOESM1_ESM.pdf]

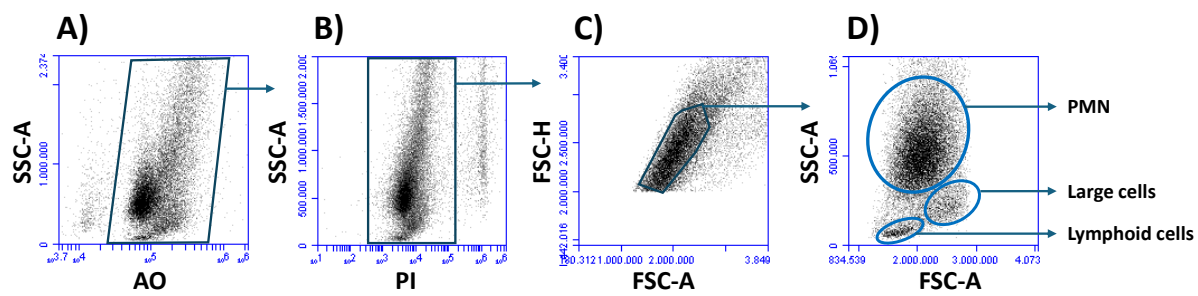

Supplement: Supplementary file 2 — Additional file 2: Additional Figure 2. Illustration of gating strategy for differential cell count in milk samples. Gating strategy to determine fractions of leukocytes among milk cells. A) Identification of nucleated cells in a PI/SSC-A density plot after staining with acridine orange (AO). B) Identification of viable, propidium iodide (PI)-negative cells in a PI/SSC-A density plot. C) Identification of single AO+/PI- cells in a FSC-area/FSC-height density plot. D) Identification of major cell populations among single AO+/PI- milk cells in a FSC-A/SSC-A density plot. PMN (polymorphonuclear leukocytes), lymphoid cells, and large cells were identified according to Mehne et al. (2010) [58], who used cell type-specific antibodies to identify polymorphonuclear neutrophils (PMN), lymphoid cell subpopulations, monocytes, and macrophages. FSC, forward scatter; SSC, side scatter. [file 12917_2024_3996_MOESM2_ESM.pdf]
